# Information Bottleneck Signal Processing and Learning to Maximize Relevant Information for Communication Receivers

**DOI:** 10.3390/e24070972

**Published:** 2022-07-14

**Authors:** Jan Lewandowsky, Gerhard Bauch, Maximilian Stark

**Affiliations:** 1 Fraunhofer Institute for Communication, Information Processing and Ergonomics, Fraunhoferstraße 20, 53343 Wachtberg, Germany; 2 Institute of Communications, Hamburg University of Technology, Eißendorfer Straße 40, 21073 Hamburg, Germany; bauch@tuhh.de (G.B.); maximilian.stark@tuhh.de (M.S.)

**Keywords:** information bottleneck, mutual information, machine learning

## Abstract

Digital communication receivers extract information about the transmitted data from the received signal in subsequent processing steps, such as synchronization, demodulation and channel decoding. Technically, the receiver-side signal processing for conducting these tasks is complex and hence causes bottleneck situations in terms of power, delay and chip area. Typically, many bits per sample are required to represent and process the received signal in the digital receiver hardware accurately. In addition, demanding arithmetical operations are required in the signal processing algorithms. A popular recent trend is designing entire receiver chains or some of their crucial building blocks from an information theoretical perspective. Signal processing blocks with very simple mathematical operations can be designed to directly maximize the relevant information that flows through them. At the same time, a strong quantization reduces the number of bits processed in the receiver to further lower the complexity. The described system design approach follows the principle of the information bottleneck method. Different authors proposed various ideas to design and implement mutual information-maximizing signal processing units. The first important aim of this article is to explain the fundamental similarities between the information bottleneck method and the functionalities of communication receivers. Based on that, we present and investigate new results on an entire receiver chain that is designed following the information bottleneck design principle. Afterwards, we give an overview of different techniques following the information bottleneck design paradigm from the literature, mainly dealing with channel decoding applications. We analyze the similarities of the different approaches for information bottleneck signal processing. This comparison leads to a general view on information bottleneck signal processing which goes back to the learning of parameters of trainable functions that maximize the relevant mutual information under compression.

## 1. Introduction

As Claude Elwood Shannon postulated in [1], the “fundamental problem of communication is that of reproducing at one point either exactly or approximately a message selected at another point”. Hence, in information theoretical terms, the most essential task of any digital communication receiver is to provide a maximum possible amount of mutual information on the transmitted data to its user. One could loosely say that the message selected at the transmitter is relevant to the receiver. Assuming that the possible transmitted messages are chosen from a finite set with certain probabilities at the transmitter, this introduces a quite intuitive understanding of a discrete relevant random variable in the communications context. The transmitted data sequence is relevant to the user of the receiver, but the latter can typically only observe a degraded version of this sequence in the form of a noisy and disturbed received signal.

Interestingly, the concept of a relevant random variable was also introduced by Tishby et al. in [2] in a very generic information theoretical setup termed the information bottleneck method. Conceptually, this method is not directly linked to the communication problem considered above. The elementary idea of the information bottleneck method is to compress an observed random variable Y to a compressed variable T using a compression rule. The compression rule is tailored to preserve relevant mutual information I(X;T), where I(X;T)≤I(X;Y). In this problem formulation, X is a chosen random variable of interest. This variable defines which features of the observation Y are relevant and should be preserved under the invoked compression. Hence, this concept can be understood as defining relevance through another variable [2]. The information bottleneck method already has numerous very successful applications, such as in image and speech processing, astronomy and neuroscience [3,4,5,6]. A comprehensive tutorial on the information bottleneck method, its applications in source coding and its connections to inference and representation learning problems can be found in [7]. In addition, a survey on the applicability and usefulness of the information bottleneck for machine learning is provided in [8].

In the past few years, the information bottleneck method gained massive popularity in the communications community. Various authors brought up different ideas to design and implement receiver subsystems and other parts of communcation systems using the information bottleneck principle of maximizing the preserved relevant information. The applications studied in the communications context lead from the design of channel output quantizers [9,10,11] over the decoding of low-density parity-check (LDPC) codes [12,13,14,15,16,17,18,19,20,21,22,23,24,25,26,27,28,29,30,31] and polar codes [32,33,34] to entire receiver chains that include channel estimation and detection [35,36,37,38]. Moreover, the information bottleneck method has been applied in joint source–channel coding, forwarding and relaying applications [39,40,41,42,43,44,45,46,47,48] and in distributed sensor networks [49,50,51,52,53] successfully. Related works with a focus on inference with the distributiveness of data among multiple nodes and network learning aspects include [54,55,56].

It needs to be noted that some of the aforementioned works do not explicitly state themselves to be instances of the information bottleneck method. However, their fundamental ideas are in line with the key principle of the preservation of relevant information under compression. Please also note that even though the references provided above are numerous, we cannot claim to have mentioned all applications of the information bottleneck method and the related principles in communications here. Facing the huge variety of research on applications of the information bottleneck method in communications above, we note that a number of techniques have been proposed to apply information bottleneck signal processing lately.

In this article, as a first contribution, we aim to give a quite general introduction to the ideas of receiver-side information bottleneck signal processing. For that purpose, we explain the general idea of the information bottleneck method and link it to the fundamental task of a communication receiver. To illustrate the applicability of the presented ideas to real-world communication receivers, we present and investigate a strongly quantized iterative receiver that is entirely designed with the information bottleneck method and compare its performance to a conventional receiver chain. The presented information bottleneck receiver implements all signal processing operations using lookup tables that are designed with an information bottleneck algorithm. Surprisingly, the presented quantized receiver that is designed with the information bottleneck method performs just as well as a double-precision reference receiver that employs state-of-the-art signal processing algorithms and uses much more costly signal processing operations.

Some very fundamental ideas of information bottleneck signal processing were presented already in 2008 for LDPC decoding by Kurkoski et al. [12]. In fact, a special focus lies on the application of information bottleneck signal processing for the coarsely quantized message passing decoding of LDPC codes in the available literature [12,13,14,15,16,17,18,19,20,21,22,23,24,25,26,27,28,29,30,31]. It needs to be appreciated that various authors have proposed very interesting ideas to solve this problem using the paradigm of maximizing the preserved relevant information under quantization with very few bits per message.

The literature describes quantized decoders that replace the classical node operations of LDPC decoders completely with lookup tables [12,14,15,16,18,20,21,23]. In addition, the hybrid min-LUT approach from [17,22] uses lookup tables only for the variable node operations and a simple arithmetical operation for the check node operations. Moreover, computational domain approaches that pair relatively simple arithmetical operations with mutual information-maximizing quantizers to implement the node operations of LDPC decoders were studied in [24,25,30,31]. Finally, the idea to learn and implement mutual information-maximizing node operations using neural networks was studied in [28,29]. We note that the design goals of the mentioned methods for LDPC decoding are similar and mainly rely on the information bottleneck idea of maximizing the preserved relevant information. However, what differs is how the mutual information-maximizing operations for the LDPC decoders are designed and implemented.

Therefore, as a second contribution, we discuss what the approaches from the literature have in common, compare them and, based on that, draw some novel conclusions on information bottleneck signal processing in general. Our conclusions break down information bottleneck signal processing to the learning of parameters of trainable functions. The parameters are tuned to maximize the relevant information under compression.

This article is structured as follows. The next section first provides an introduction to the information bottleneck method and its application in coarsely quantized information bottleneck signal processing units. A quite general comparison of the information bottleneck method and a communication system is used to explain the applicability of the information bottleneck method for the design of quantized communication receivers. Afterwards, we substantiate the presented ideas by developing and analyzing a particular information bottleneck receiver structure for an LDPC-encoded transmission over a fading channel. This receiver implements all signal processing operations designed with the information bottleneck method using lookup tables.

Section 3 then starts by recalling and comparing other approaches to information bottleneck signal processing for LDPC decoders from the literature that do not use lookup tables. From this analysis, we draw the conclusion that a unified view on lookup table-based and other approaches is considering information bottleneck signal processing as learning the optimum parameters of trainable functions that maximize the relevant information. Finally, Section 4 summarizes and concludes this article.

## 2. The Information Bottleneck Method and Coarsely Quantized Information Bottleneck Signal Processing

This section gives an overview of the information bottleneck method and explains its connection to the fundamental design purpose of communication receivers (i.e., extracting relevant information on the transmitted data from the received signal). From that, the idea of information bottleneck signal processing is derived and explained using several examples.

### 2.1. The Information Bottleneck Method

**Figure 1:**
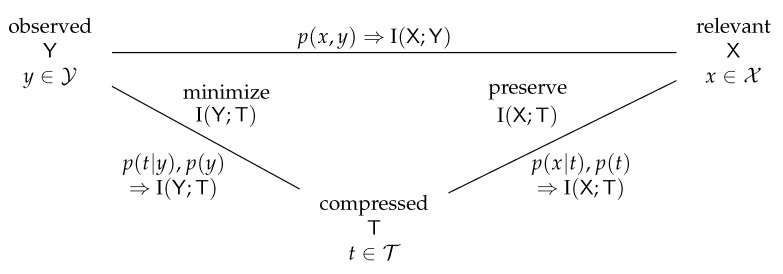
Illustration of the information bottleneck method. The variables X,Y and T form a Markov chain X→Y→T and are termed the relevant, observed and compressed variables, respectively. The fundamental principle is to minimize I(Y;T) while preserving I(X;T).

The information bottleneck method is a quite generic information theoretical setup that was introduced by Tishby et al. in [2]. The basic setup consists of three discrete random variables X, Y and T with realizations x∈X,y∈Y and t∈T. These variables follow the Markov relation X→Y→T and interact as illustrated in Figure 1 and explained in the following.

The random variable Y is considered to be observed and, therefore, termed the observed random variable in the information bottleneck problem setup. The baseline model is that observing the realizations y∈Y of Y could deliver information that could be classified into relevant and irrelevant information. In order to define which features of Y are considered relevant, the so-called relevant random variable X is introduced. As a result, the relevance is defined through another variable, and the relevant information that X and Y share is

(1) $$I\left(X;Y\right)=\sum_{x\in X}\sum_{y\in Y}p\left(x,y\right)\log\frac{p(x,y)}{p(x)p(y)}=D_{\mathrm{KL}}\left\{p(x,y)\;|\;p(x)p(y)\right\},$$

where p(x,y) is the joint distribution and p(x) and p(y) are the marginal distributions of X and Y, respectively. $D_{\mathrm{KL}}\left\{.\;|\;.\right\}$ in Equation (1) is the Kullback–Leibler divergence.

The aims of the information bottleneck method are now twofold:

1. Conduct a lossy compression of the realizations y∈Y to a compressed realization t∈T to yield a compact compressed representation T of the observation Y. The information theoretical notion of such a compression is the minimization of the compression information I(Y;T) (i.e., the transmission rate, relating to rate–distortion theory).
2. While conducting the compression mentioned above, preserve the relevant information I(X;T)≤I(X;Y).

The goals mentioned above are contradictory. Typically, a strong compression will limit the possibility to keep the preserved relevant information I(X;T) above a desired lower bound. Similarly, aiming for a certain minimum amount of I(X;T)≤I(X;Y) typically provides a lower bound to I(Y;T). As a result, an optimum rule to compress Y onto T in the information bottleneck sense describes a trade-off between achieving a minimum possible compression information I(Y;T) that also allows keeping the preserved relevant information I(X;T) above a desired minimum level.

In technical terms, the compression rule that maps Y onto T is described as a conditional probability distribution p(t|y). This conditional distribution provides the probabilities of a certain t∈T for a given y∈Y and hence describes a possibly stochastic mapping of *y* onto *t*.

The information bottleneck problem can be understood as the optimization problem of finding a suitable conditional probability distribution p(t|y) with the desired characteristics of minimizing I(Y;T) while preserving I(X;T) for the Markov chain X→Y→T. Tishby et al. proposed finding an optimum mapping p(t|y) using the Lagrange method in [2] and introduced the Lagrangian

(2) L(p(t|y))=I(Y;T)−βI(X;T)

which has to be minimized over the set of all valid conditional probability distributions p(t|y). The Lagrangian multiplier β≥0 is a trade-off parameter that allows tuning the aforementioned trade-off between the compression and preservation of relevant information. For β=0, the focus is only on compression, and the preservation of I(X;T) is not taken into account, while β→+∞ aims to maximize the preserved relevant information.

In [2], Tishby et al. also derived a set of equations characterizing p(t|y),p(t) and p(x|t) such that

(3) $$\begin{array}{ccc} p(t|y) & = & \frac{p(t)}{Z(y,\beta )}\exp\left(-\beta D_{\mathrm{KL}}\left\{p(x|y)\;|\;p(x|t)\right\}\right) \end{array}$$

(4) $$\begin{array}{ccc} p(t) & = & \sum_{y\in Y}p\left(t|y\right)p\left(y\right) \end{array}$$

(5) $$\begin{array}{ccc} p(x|t) & = & \frac{1}{p(t)}\sum_{y\in Y}p\left(t|y\right)p\left(x,y\right), \end{array}$$

where Z(y,β) is a normalization function that guarantees that p(t|y) is a valid conditional distribution.

In general, the optimization problem of finding a p(t|y) solution that minimizes the Lagrangian from Equation (2) is neither concave nor convex [2]. Nevertheless, the mentioned equations (Equations (3–5)) naturally suggest an iterative algorithm termed the iterative information bottleneck algorithm to obtain p(t|y) for a given joint distribution p(x,y), a trade-off parameter β and an intended cardinality |T| of the compression variable T that is also described in [2]. This algorithm provably converges to at least a local minimum of the Lagrangian in Equation (2). Details on its convergence are discussed in [3]. In addition to the iterative information bottleneck algorithm from [2], many other information bottleneck algorithms appeared in the literature (e.g., [57,58]). These algorithms can be understood as the work horses of the information bottleneck method, as they can determine the compression mapping p(t|y) for a given p(x,y), β and a desired cardinality |T|. The information bottleneck algorithms also deliver the distributions p(x|t) and p(t), and thus p(x,t)=p(x|t)p(t) according to Equations (4) and (5) as side products.

**Figure 2:**
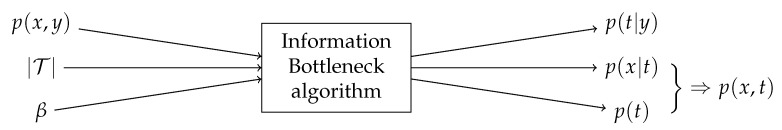
Overview of the inputs taken and the outputs delivered by an information bottleneck algorithm.

Figure 2 provides an overview of the inputs taken and the outputs delivered by an information bottleneck algorithm.

An important notion of the resulting conditional probability distribution p(t|y) is that it clusters the event space Y of the observed random variable Y into clusters $Y_{t}$, t∈{0,1,…,|T|−1}. This concept covers deterministic mappings of *y* onto *t* (i.e., p(t|y)∈{0,1}∀(y,t)) that result in a hard clustering of Y and also probabilistic mappings that describe a soft clustering. For the latter, the realizations *y* can be contained in various clusters $Y_{t}$ with different probabilities.

In the very important special case of aiming to preserve a maximum desired amount of I(X;T) (i.e., β→+∞ for a given cardinality |T|), the clustering of Y described by p(t|y) becomes a hard clustering [58]. In this case, it is easy to limit the compression information I(Y;T) by a proper choice of the cardinality |T| of the compressed representation. In the context of this article, this cardinality determines the number of bits required to process the compressed realizations t∈T in the digital hardware of a communication receiver. The remainder of this article will only focus on deterministic compression mappings, as these are most practical to be applied in digital communication receivers.

It will be important in the following that a deterministic mapping p(t|y) can also be interpreted as a deterministic input/output relation of a system with input y∈Y and output t∈T. Such a system can be implemented, for example, as a lookup table that holds the respective t∈T for each possible y∈Y if the input space Y has a manageable cardinality. As we will discuss later in Section 3, the lookup table interpretation is just one possibility for implementing deterministic information bottleneck compression mappings in communication receivers. We want to mention here that we only formally consider discrete observed variables Y in this article. The reason for this is that every digital communication receiver needs to quantize the continuous received signal with a limited number of bits per sample before conducting further digital signal processing steps, as will be explained in more detail later.

Before we continue, an important concept from [35] shall be revisited. As the mutual information relation between X, Y and T in the information bottleneck method is quite abstract, it is reasonable to introduce a compact graph notation that allows one to visualize the intended mutual information relations between the variables involved in an information bottleneck problem. In [35], information bottleneck graphs were introduced for that purpose.

**Figure 3:**
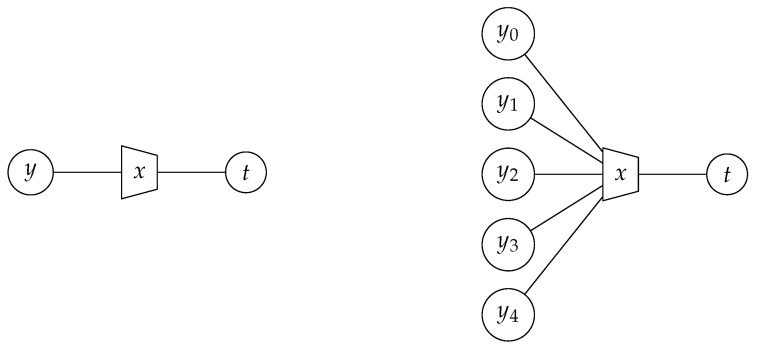
Examples of information bottleneck graphs. The trapezoid nodes correspond to the compression mappings p(t|y) and $p(t|y_{0},y_{1},y_{2},y_{3},y_{4})$, respectively. Both are designed to preseve I(X;T).

Information bottleneck graphs are extended factor graphs that aim to compactly visualize the intended mutual information relations of the information bottleneck method. In an information bottleneck graph, a compression mapping p(t|y) designed with an information bottleneck algorithm is visualized as a trapezoid node that is labeled with the respective relevant random variable. The compressed variable is connected to the shortest side of the trapezoid. The other connected variables form the observation. This concept allows one to cover compression mappings with a single scalar input *y* or multiple scalar inputs $y_{n},\;n\in \left\{0,1,\ldots ,N-1\right\}$. Figure 3 shows an example for a compression mapping p(t|y) with one scalar input on the left. On the right of the figure, an example for a compression mapping $p(t|y_{0},y_{1},y_{2},y_{3},y_{4})$ with five scalar inputs is shown. A compression mapping $p(t|y_{0},y_{1},\ldots ,y_{N-1})$ with *N* scalar inputs can equivalently be seen as a compression mapping p(t|y) for a row vector $y=[y_{0},y_{1},\ldots ,y_{N-1}]$. In this case, the observed random variable Y is a random vector, but the principle of the information bottleneck method remains unchanged, and the compression mapping p(t|y) can still be designed with the available information bottleneck algorithms. Problems arise, however, for a large number of inputs *N*, as the number of possible conditions y might grow massively with *N*. Nonetheless, Figure 3 illustrates that the intended information relations can be visualized very compactly in information bottleneck graphs. Information bottleneck graphs will be used extensively in Section 2.3.

### 2.2. General View on Information Bottleneck Signal Processing for Receiver Design

**Figure 4:**
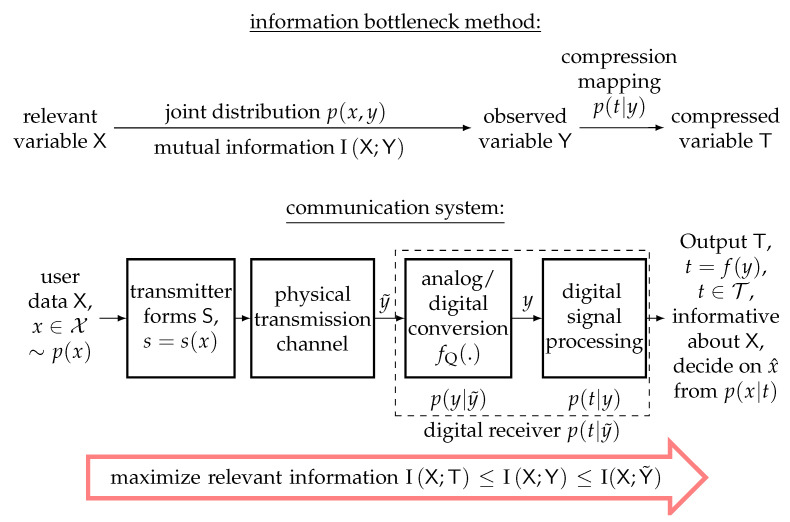
Illustration of the information bottleneck design idea of a communication system. The digital communication receiver shall be designed such that the relevant information I(X;T) is maximized from end to end.

Conceptually, the purpose of the information bottleneck method is intuitively related to the most famous fundamental problem of communications cited at the beginning. In order to explain this connection, Figure 4 compares the information bottleneck method at the top with a model view of a communication system at the bottom.

As shown in the figure and discussed above, the transmitted user data X can be considered to be relevant, and the task is to estimate the realization *x* after the transmitter has transformed it into a transmit signal s(x) to prepare it for the transmission over a physical transmission channel. The transmission of s(x) then results in the received realization $\tilde{y}$, which is typically continuous.

As shown in Figure 4, we assume that $\tilde{y}$ is quantized to a discrete representation *y* by an analog-to-digital converter with the quantization function $f_{Q}\left(.\right)$ in the receiver (i.e., $y=f_{Q}\left(\tilde{y}\right)$). The quantized representation *y* is then used for further digital signal processing steps to estimate *x*. Digital communication receivers often have to process huge blocks of the received samples at once due to the transmitter-side channel coding and modulation techniques that spread information on a transmitted bit *x* over a huge block of transmitted symbols. To obtain a simple notation that is coherent with the information bottleneck notation used above, we do not explicitly highlight the fact the variables X,Y and T could be random vectors in the communication system from Figure 4. However, one should keep in mind that a communication receiver typically estimates a vector x of transmitted bits from a long vector $\tilde{y}$ of channel observations. In this case, the application of $f_{Q}\left(.\right)$ has to be understood as an element-wise application (i.e., $y_{k}=f_{Q}\left({\tilde{y}}_{k}\right)$, with the time index *k* enumerating subsequent samples).

The amount of information present about X in the quantized received signal Y is bounded by the capacity of the quantized output transmission channel p(y|x) at the upper bound, which is given by

(6) $$C=\max_{p(x)}I\left(X;Y\right)\leq \max_{p(x)}I\left(X;\tilde{Y}\right).$$

Hence, the task of the communication receiver is to extract the information on the relevant X and to provide the best possible estimate $\hat{x}$ at its output. Clearly, such an estimate can be obtained from a receiver output variable T that fulfils I(X;T)→max. As a result, the further receiver-side digital signal processing modeled by p(t|y) should maximize I(X;T). Then, p(x|t) can provide an estimate of the transmitted data *x*, for example, using the maximum a-posteriori criterion:

(7) $$\hat{x}=\arg\max_{x}p\left(x|t\right).$$

Please note that p(x|t) is inherently obtained when the information bottleneck method is applied according to Equation (4). This already indicates that it is possible to estimate *x* based on a compressed representation *t* of the quantized received signal *y*.

An interesting fact in this context is that in information theoretical terms, all signal processing of Y can only preserve or lower the information on X as a direct result of the data processing inequality [59] (i.e., I(X;T)≤I(X;Y)). Due to the typically very advanced channel coding and modulation steps conducted at the transmitter, obtaining an estimate $\hat{x}$ for *x*, for example, based on the straightforward application of the maximum likelihood criterion via the equation

(8) $$\hat{x}=\arg\max_{x}p\left(y|x\right)$$

is often prohibitively complex. The reason for this is that the transmitter spreads information on the user data *x* over huge blocks of transmitted symbols to protect it against transmission errors, as already mentioned above. In addition, it might include control data in the transmitted data stream to simplify the receiver-side detection process, for example, for channel estimation. As a result, the digital communication receiver has to aggregate the information on the user data X by undoing the transmitter-side encoding and modulation process using suitable algorithms composed in a signal processing chain to process the received *y*.

In order to overcome the complexity of a straightforward maximum likelihood sequence detection, it is common practice to separate and distribute receiver-side signal processing tasks such as synchronization, channel estimation, detection and channel decoding over concatenated signal processing blocks that exchange messages or signals. Unfortunately, the performance of such signal processing algorithms still relies on sufficient precision. The number of bits per sample has to be large enough to adequately represent the received signal in the digital hardware without a degrading quantization distortion. In addition, the required signal processing operations are often computationally demanding.

It is well known that a coarse quantization (i.e., a low resolution of the analog-to-digital converter) can help to reduce the implementation efforts of communication receivers, as it directly reduces the number of bits needed to process the signal in the digital hardware. In addition, simplified arithmetical operations can reduce the implementation efforts of optimum or close-to-optimum signal processing algorithms. However, both typically result in performance degradations.

Therefore, the fundamental idea of information bottleneck signal processing is to build subptimum receiver components that, despite being strongly quantized, are designed to preserve the maximum possible amount of relevant information and only use simple arithmetical operations. This design idea inherently minimizes the inevitable performance degradation resulting from the quantization and from using potentially suboptimal signal processing operations.

Information bottleneck signal processing has already been applied to various receiver-side signal processing tasks in the past, such as quantized detection and channel estimation [35,36,37,38]. In the following, we will analyze a receiver chain that applies information bottleneck signal processing extensively. In this receiver, we consider the mutual information preserving input/output relations of p(t|y) to be implemented as lookup tables that replace the conventional signal processing operations.

### 2.3. An Example of Information Bottleneck Receiver Design with Iterative Detection and Decoding

In this section, we illustrate the idea of information bottleneck signal processing by considering data transmission with binary phase shift keying (BPSK) modulation over a frequency flat block fading channel. The presented study continues and extends the investigation from our conference publication [60].

We consider data transmission over a frequency-flat block fading channel. The complex-valued continuous received signal at time instance *k* in the symbol clock is given by

(9) $${\tilde{y}}_{k}=\tilde{h}s_{k}+{\tilde{n}}_{k}={\tilde{y}}_{k}^{\mathrm{re}}+j{\tilde{y}}_{k}^{\mathrm{im}},$$

where $\tilde{h}$ is a complex channel coefficient from a zero-mean circularly complex Gaussian process that is modelled as a constant for *B* symbol durations and $s_{k}\in \left\{-1,+1\right\}$ is the transmitted BPSK symbol. The noise sample ${\tilde{n}}_{k}$ is also a realization from a complex zero-mean white Gaussian process with variance $\sigma_{\tilde{n}}^{2}$. Please note that in our notation, we use a tilde to indicate that a signal has not been quantized and hence is continuous, and the superscripts re and im distinguish real and imaginary parts, respectively.

**Figure 5:**
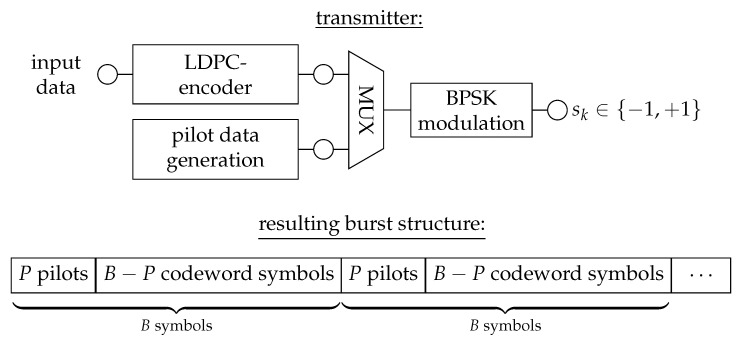
Overview of the considered transmitter. *P* pilot bits are multiplexed into a length $N^{\mathrm{LDPC}}>>B$ LDPC codeword periodically with a distance of B−P bits, and the resulting data stream is modulated using BPSK modulation.

For clarity, Figure 5 provides an overview of the considered transmitter that is explained in the following.

The transmitter transmits codewords from a regular LDPC code with a codeword length $N^{\mathrm{LDPC}}>>B$ over the channel using BPSK modulation. In order to simplify the receiver-side detection and decoding, the transmitter multiplexes *P* known pilot symbols into a transmitted codeword with a distance of B−P symbols such that each block of *B* symbols weighted with the same channel coefficient $\tilde{h}$ starts with a few known pilot symbols. The resulting burst structure is also sketched at the bottom of Figure 5. These pilots shall serve as a reference signal to enable a simple receiver-side channel estimation.

**Figure 6:**
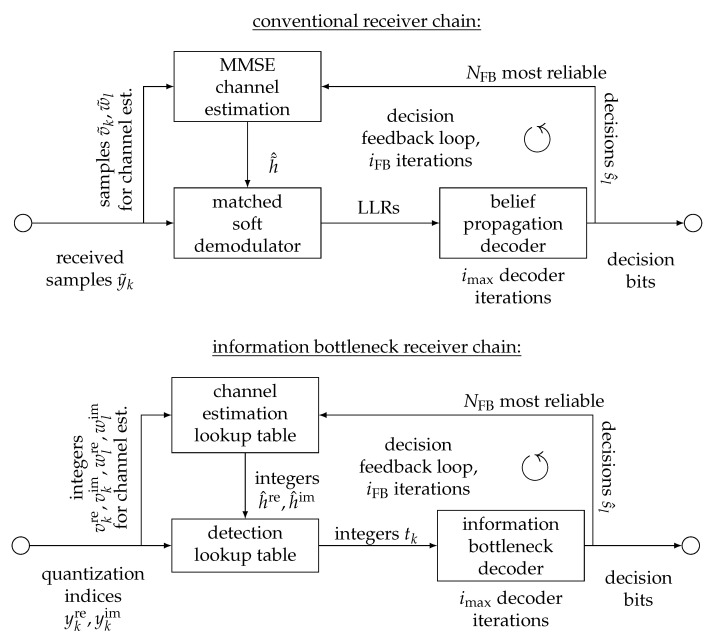
Conventional and information bottleneck receiver chains for LDPC-encoded data transmission over a frequency-flat fading channel. The conventional receiver uses quasi-continuous received samples ${\tilde{y}}_{k}$ with double-precision and processes them in state-of-the-art detection and decoding algorithms. The information bottleneck receiver works on quantization indices and implements all signal processing using information bottleneck lookup tables.

Figure 6 shows a conventional receiver structure for the considered transmitter that uses classical signal processing operations and does not employ any quantization beyond the numerical limits of double-precision at the top. This receiver shall serve as a reference receiver that illustrates the performance of the considered data transmission scheme if no quantization is applied at all. It initially employs minimum mean squared error (MMSE) channel estimation based on the inserted pilot symbols. Based on the obtained channel estimate, it then performs a matched soft demodulation. The term matched reflects that the demodulator takes into account the uncertainty of the estimated channel coefficient. The demodulator then delivers log-likelihood ratios (LLRs) and provides them to a belief propagation decoder for the decoding of the LDPC code. This decoder performs $i_{\max}$ decoding iterations. After one such decoding round, the receiver has estimates of the transmitted codeword bits. The reliability of the different codeword bits can be judged by analyzing the absolute magnitudes of the decision LLRs from the decoder. In order to further improve the detection and decoding quality, the receiver then iteratively employs decision feedback (FB) of the $N_{\mathrm{FB}}$ most reliable decision bits within a block to virtually enlarge the number of available pilot symbols used for channel estimation. The considered receiver implements a hard-decision turbo channel estimation scheme. Its decision feedback loop is repeated $i_{\mathrm{FB}}$ times, and then the final decision bits are provided.

Underneath the conventional receiver chain in Figure 6, the proposed information bottleneck receiver chain is depicted. At first glance, both receiver chains look similar. The most important difference is, however, that the information bottleneck receiver chain does not have access to the continuous received samples ${\tilde{y}}_{k}$. Instead, it only processes quantization indices from a coarse channel output quantizer, which quantizes the real and imaginary parts of the received signal to quantization indices (i.e., $y_{k}^{\mathrm{re}},y_{k}^{\mathrm{im}}\in \left\{0,1,\ldots ,2^{q}-1\right\}$). Based on these observed quantization indices, the rest of the signal processing chain for channel estimation, detection and LDPC decoding can also be developed using the information bottleneck method. This will be explained in detail in the following.

#### 2.3.1. Information Bottleneck Channel Estimation

In the considered conventional receiver chain, the task of the channel estimation is to obtain a reliable estimate $\hat{\tilde{h}}$ of $\tilde{h}$ from the knowledge of *P* pilot symbols and their respective received samples. We denote these received samples by ${\tilde{v}}_{0},{\tilde{v}}_{1},\ldots ,{\tilde{v}}_{P-1}$. The MMSE channel estimate is then given by

(10) $$\hat{\tilde{h}}=\frac{1}{P+\sigma_{\tilde{n}}^{2}/\sigma_{\tilde{h}}^{2}}\sum_{k=0}^{P-1}{\tilde{v}}_{k}s_{k}^{p},$$

where $s_{k}^{p}\in \left\{-1,+1\right\}$ are the known pilot symbols.

It is obvious, that the evaluation of Equation (10) requires high-precision arithmetic. In a digital receiver, a high quantization resolution is required such that the quantized received samples adequately approximate the continuous ${\tilde{v}}_{k}$ to obtain a good estimate with high accuracy. As mentioned above, we assume that the conventional reference receiver is implemented using double floating-point precision and therefore does not suffer from any mentionable quantization loss.

In the information bottleneck receiver, however, the aim is to directly process the *q*-bit integer indices $v_{k}^{\mathrm{re}}=f_{Q}\left({\tilde{v}}_{k}^{\mathrm{re}}\right)$ and $v_{k}^{\mathrm{im}}=f_{Q}\left({\tilde{v}}_{k}^{\mathrm{im}}\right)$. The function $f_{Q}\left(.\right)$ describes a channel output quantizer that is also designed to preserve the maximum relevant information with the information bottleneck method as explained in [35]. Please note that the design of the channel output quantizer $f_{Q}\left(.\right)$ is not discussed in detail in this article. However, we want to mention that it requires handling continuous observed random variables with the information bottleneck method, which has not been discussed in this article so far. A simple method to deal with continuous observations for the quantizer design problem is approximating the continuous variables as very finely quantized discrete variables. More details on the quantizer design with a desired maximum preservation of relevant information can be found in [35,61].

The quantization function $f_{Q}\left(.\right)$ conducts threshold decisions on the real and imaginary parts of the received signal ${\tilde{y}}_{k}$. It delivers quantization indices from the set $\left\{0,1,\ldots ,2^{q}-1\right\}$. This reflects an analog-to-digital conversion with *q* bits per sample in the real and imaginary parts, respectively. The obtained unsigned integers neither approximate the continuous received samples nor can they be used to obtain a channel estimate with an arithmetic rule such as that in Equation (10).

Essentially, the task of channel estimation is to extract information on the unknown channel coefficient $\tilde{h}$ from *P* pairs of integers $\left(v_{k}^{\mathrm{re}},v_{k}^{\mathrm{im}}\right)$ that correspond to the coarsely quantized received samples for the pilot symbols. Please note that due to the considered channel model and the fact that the pilot symbols are BPSK symbols, it is possible to handle $v_{k}^{\mathrm{re}}$ and $v_{k}^{\mathrm{im}}$ independently. In the following, the signal processing of $v_{k}^{\mathrm{im}}$ will not be discussed, as it is equivalent to that of $v_{k}^{\mathrm{re}}$.

Note that the quantizer $f_{Q}\left(.\right)$ leads to the fact that up to the distortion caused by the receiver noise and the influence of $s_{k}\in \left\{-1,+1\right\}$, the real part of the continuous channel coefficient $\tilde{h}$ is also observed in a quantized manner. Therefore, the integer $h^{\mathrm{re}}$ is the index of the quantization region the continuous ${\tilde{h}}^{\mathrm{re}}$ falls into; that is, $h^{\mathrm{re}}=f_{Q}\left({\tilde{h}}^{\mathrm{re}}\right)$. From an information theoretical perspective, the task of a pilot-based channel estimator is to map the unsigned integers $v_{0}^{\mathrm{re}},v_{1}^{\mathrm{re}},\ldots ,v_{P-1}^{\mathrm{re}}$ onto ${\hat{h}}_{\mathrm{FW}}^{\mathrm{re}}$ such that the mutual information $I\left({\hat{H}}_{\mathrm{FW}}^{\mathrm{re}};H^{\mathrm{re}}\right)$ is maximized. Please note that we add the index forward (FW) to distinguish the channel estimation from the one conducted in the feedback (FB) iterations, which will be discussed later.

An intuitive approach to designing an information bottleneck equivalent to the pilot-based channel estimation in the forward path of the conventional receiver is to design an information bottleneck compression mapping $p\left({\hat{h}}_{\mathrm{FW}}^{\mathrm{re}}|v_{0}^{\mathrm{re}},v_{1}^{\mathrm{re}},\ldots ,v_{P-1}^{\mathrm{re}}\right)$ which aims to maximize $I\left({\hat{H}}_{\mathrm{FW}}^{\mathrm{re}};H^{\mathrm{re}}\right)$. However, designing and implementing this compression mapping as a lookup table would typically result in prohibitive complexity, since the cardinality of the observation random variable $\left(V_{0}^{\mathrm{re}},V_{1}^{\mathrm{re}},\ldots ,V_{P-1}^{\mathrm{re}},\right)$ is $2^{q\cdot P}$. For example, with q=5 for bit quantization and P=6 pilot symbols, this would result in a lookup table with more than $10^{9}$ entries. This cardinality exceeds the afordable runtime and space complexities of all available information bottleneck algorithms, as these algorithms typically have to handle a dense matrix representation of p(x,y) that consists of |X||Y| real numbers. Moreover, the resulting lookup table would be prohibitively large.

**Figure 7:**
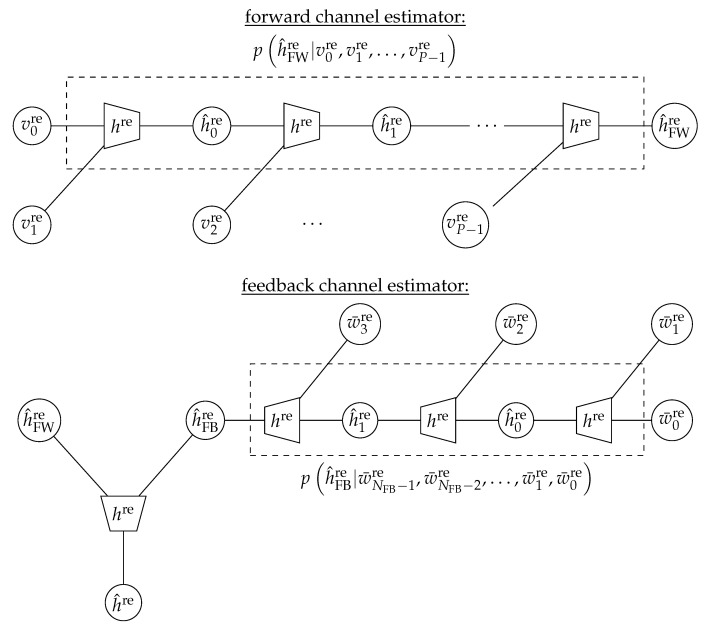
Information bottleneck graphs of the forward and feedback channel estimation schemes for the information bottleneck receiver. The upper part of the figure shows the forward channel estimator consisting of P−1 two-input information bottleneck compression mappings. The lower part shows the feedback channel estimator with a similar structure to process $N_{\mathrm{FB}}=4$ inputs.

In order to cope with the resulting complexity, a particularly useful feature of information bottleneck compression mappings is that they can be concatenated to reduce the complexity. Such a concatenation is shown in an information bottleneck graph in the upper part of Figure 7 for the considered channel estimation scheme for the pilot-based forward channel estimation. As shown, the task of processing *P* quantized samples is split into a series of P−1 concatenated compression mappings, each of which processes two inputs. This way, the space complexity of the component compression mappings that we implement as lookup tables is drastically reduced to a concatenation of lookup tables with $2^{2q}+\left(P-2\right)2^{q^{\mathrm{ce}}+q}$ entries, where $q^{\mathrm{ce}}$ denotes the number of bits needed to represent the intermediate results ${\hat{h}}_{m}^{\mathrm{re}}$ from Figure 7. Please note that this number of bits is a design choice, and it can be adjusted by the choice of the cardinality of the compression variable of the information bottleneck algorithm that is used. For simplicity, all compression mappings shall use the same output bit width $q^{\mathrm{ce}}$ such that all intermediate results ${\hat{h}}_{m}^{\mathrm{re}}$ and the final output ${\hat{h}}_{\mathrm{FW}}^{\mathrm{re}}$ are from the same set $\left\{0,1,\ldots ,2^{q^{\mathrm{ce}}}-1\right\}$. With $q^{\mathrm{ce}}=5$ and P=6 pilot symbols, as considered before, the overall size of the lookup tables to implement the concatenated scheme from Figure 7 is reduced from roughly $10^{9}$ to 5120.

All compression mappings appearing inside the forward channel estimator from Figure 7 preserve information on the same variable $H^{\mathrm{re}}$. The design of the compression mappings from Figure 7 requires feeding the joint distributions

(11) $$p\left(h^{\mathrm{re}},v_{0}^{\mathrm{re}},v_{1}^{\mathrm{re}}|s_{0}^{p},s_{1}^{p}\right)=p\left(h^{\mathrm{re}},v_{0}^{\mathrm{re}}|s_{0}^{p}\right)p\left(h^{\mathrm{re}},v_{1}^{\mathrm{re}}|s_{1}^{p}\right)\frac{1}{p\left(h^{\mathrm{re}}\right)}$$

and

(12) $$\begin{array}{c} p\left(h^{\mathrm{re}},{\hat{h}}_{m-1}^{\mathrm{re}},v_{m+1}^{\mathrm{re}}|s_{0}^{p},s_{1}^{p},\ldots ,s_{m+1}^{p}\right)=\\ p\left(h^{\mathrm{re}},{\hat{h}}_{m-1}^{\mathrm{re}}|s_{0}^{p},s_{1}^{p},\ldots ,s_{m}^{p}\right)p\left(h^{\mathrm{re}},v_{m+1}^{\mathrm{re}}|s_{m+1}^{p}\right)\frac{1}{p\left(h^{\mathrm{re}}\right)}\;\mathrm{for}\;m\geq 1. \end{array}$$

to an information bottleneck algorithm.

One question that is still open is how to obtain the joint distributions $p\left(h^{\mathrm{re}},v_{m}^{\mathrm{re}}|s_{m}^{p}\right)$ needed to evaluate Equations (11) and (12). Recall that $h^{\mathrm{re}}$ and $v_{m}^{\mathrm{re}}$ are quantization indices of ${\tilde{h}}^{\mathrm{re}}$ and ${\tilde{v}}_{m}^{\mathrm{re}}$, respectively. Hence, $h^{\mathrm{re}}=f_{Q}\left({\tilde{h}}^{\mathrm{re}}\right)$ and $v_{m}^{\mathrm{re}}=f_{Q}\left({\tilde{v}}_{m}^{\mathrm{re}}\right)$, where $f_{Q}\left(.\right)$ characterizes the threshold decisions of the channel output quantizer. The quantizer $f_{Q}\left(.\right)$ for ${\tilde{h}}^{\mathrm{re}}$ and ${\tilde{v}}^{\mathrm{re}}$ can also be described by $p\left(h^{\mathrm{re}}|{\tilde{h}}^{\mathrm{re}}\right)$ and $p\left(v_{m}^{\mathrm{re}}|{\tilde{v}}_{m}^{\mathrm{re}}\right)$. This allows expressing the joint distribution $p\left(h^{\mathrm{re}},v_{m}^{\mathrm{re}}|s_{m}^{p}\right)$ as

(13) $$p\left(h^{\mathrm{re}},v_{m}^{\mathrm{re}}\right|s_{m}^{p})=\int_{-\infty }^{+\infty }\int_{-\infty }^{+\infty }p\left({\tilde{h}}^{\mathrm{re}},{\tilde{v}}_{m}^{\mathrm{re}}|s_{m}^{p}\right)p\left(h^{\mathrm{re}}|{\tilde{h}}^{\mathrm{re}}\right)p\left(v_{m}^{\mathrm{re}}|{\tilde{v}}_{m}^{\mathrm{re}}\right)\;d\;{\tilde{h}}^{\mathrm{re}}\;d\;{\tilde{v}}_{m}^{\mathrm{re}}.$$

In this integral, the factors $p\left(h^{\mathrm{re}}|{\tilde{h}}^{\mathrm{re}}\right)$ and $p\left(v_{m}^{\mathrm{re}}|{\tilde{v}}_{m}^{\mathrm{re}}\right)$ gather all the probability masses of the continuous ${\tilde{h}}^{\mathrm{re}}$ and ${\tilde{v}}_{m}^{\mathrm{re}}$, which are mapped onto the same pair $\left(h^{\mathrm{re}},v_{m}^{\mathrm{re}}\right)$ by application of the channel output quantizer on these variables. Therefore, they determine the integration area for a particular pair $\left(h^{\mathrm{re}},v_{m}^{\mathrm{re}}\right)$. The distribution $p\left({\tilde{h}}^{\mathrm{re}},{\tilde{v}}_{m}^{\mathrm{re}}|s_{m}^{p}\right)$ for the considered channel model is a multivariate Gaussian distribution. Both components have a zero mean. As a result, the multivariate Gaussian distribution $p\left({\tilde{h}}^{\mathrm{re}},{\tilde{v}}_{m}^{\mathrm{re}},|s_{m}^{p}\right)$ is fully characterized by the covariance matrix

(14) $$C_{{\tilde{H}}^{\mathrm{re}},{\tilde{V}}_{m}^{\mathrm{re}}|S_{m}^{p}}=\frac{\sigma_{\tilde{h}}^{2}}{2}\left[\begin{array}{cc} 1 & s_{m}^{p}\\ s_{m}^{p} & 1 \end{array}\right]+\frac{\sigma_{\tilde{n}}^{2}}{2}\left[\begin{array}{cc} 0 & 0\\ 0 & 1 \end{array}\right].$$

The integral from Equation (13) with the considered Gaussian distributions and covariance matrices characterized by Equation (14) is easily solved numerically using the method described in [62]. This allows one to obtain the joint distributions $p\left(h^{\mathrm{re}},v_{m}^{\mathrm{re}}\right|s_{m}^{p})$ and hence construct the concatenated structure of information bottleneck compression mappings from the top of Figure 7 using Equations (11) and (12). We apply the KL-means algorithm from [57] to construct the proposed information bottleneck channel estimator, as this algorithm can be highly parallelized and is very fast.

The design process of the information bottleneck channel estimator delivers the joint distribution $p\left({\hat{h}}^{\mathrm{re}},h^{\mathrm{re}}\right)=p\left(h^{\mathrm{re}}|{\hat{h}}^{\mathrm{re}}\right)p\left({\hat{h}}^{\mathrm{re}}\right)$, which has to be used further to construct an information bottleneck detection lookup table. Its design is explained in the next subsection.

A very similar concept of information bottleneck channel estimation can also be used in the feedback iterations of the iterative information bottleneck receiver structure from Figure 6 to implement a turbo-like channel estimation scheme. After decoding the LDPC code, the receiver can identify the most reliable bit decisions ${\hat{s}}_{l}$ and their corresponding received samples $w_{l}^{\mathrm{re}}$.

Let $w_{l}^{\mathrm{re}}$, $l\in \{0,1,\ldots ,N_{\mathrm{FB}}-1\}$ denote the quantized integer indices $y_{k}^{\mathrm{re}}$, which correspond to the most reliable bit decisions in the decoded codeword. Moreover, let ${\hat{s}}_{l}$ denote the respective hard decision BPSK symbols corresponding to the respective decision bits. The task of an information bottleneck feedback channel estimator is to extract the information on $h^{\mathrm{re}}$ from ${\hat{s}}_{l}$ and $w_{l}^{\mathrm{re}}$. Please note that the transformation

(15) $${\bar{w}}_{l}^{\mathrm{re}}=\left\{\begin{array}{cc} w_{l}^{\mathrm{re}} & {\hat{s}}_{l}=+1\\ 2^{q}-w_{l}^{\mathrm{re}}-1 & {\hat{s}}_{l}=-1. \end{array}\right.$$

undoes the influence of ${\hat{s}}_{l}$ and makes all ${\bar{w}}_{l}^{\mathrm{re}}$ look like quantization indices received for the transmitted symbol $s_{l}=+1$ if the decision ${\hat{s}}_{l}$ is correct.

The integers ${\bar{w}}_{l}^{\mathrm{re}}$ can be used in the feedback channel estimation scheme shown in the bottom part of Figure 7. The design of the concatenated compression mappings is completely equivalent to the one for the forward channel estimator above, with the only difference being that one assumes the transmitted symbol to be $s_{l}=+1$ due to the transformation from Equation (15). In fact, this transformation is a simple way to make the feedback channel estimation lookup tables independent of the symbol decision and thus keep them minimal in size. However, we want to mention that this method only works for BPSK modulation. Higher-order modulation schemes are not studied in this article. The presented information bottleneck channel estimation could be implemented for higher-order modulation schemes equivalenty, but this might also require feeding the symbol decisions ${\hat{s}}_{l}$ to the feedback channel estimation lookup tables.

The final compression mapping $p({\hat{h}}^{\mathrm{re}}|{\hat{h}}_{\mathrm{FW}}^{\mathrm{re}},{\hat{h}}_{\mathrm{FB}}^{\mathrm{re}})$ combines the relevant information on $h^{\mathrm{re}}$ from the forward and feedback channel estimator lookup tables to finally deliver the integer ${\hat{h}}^{\mathrm{re}}$ that is highly informative about $h^{\mathrm{re}}$. The obtained ${\hat{h}}^{\mathrm{re}}$ can be used for detection in the next step.

#### 2.3.2. Information Bottleneck Detection

In a conventional receiver with high precision, the obtained MMSE channel estimate $\hat{\tilde{h}}$ from Equation (10) can be used for soft demodulation. The LLRs of the soft demodulator are given by

(16) Lch(sk)=11+1P+σn˜2/σh˜2·4Reh˜^*r˜kσn˜2,

where Re{.} denotes the real part of a complex number and $.^{*}$ denotes the conjugate complex. Using these channel LLRs, it is easy to apply the LLR-based belief propagation decoding algorithm in the conventional receiver at the top of Figure 6.

When the information bottleneck channel estimator from the preceding section is applied, it delivers the pair $\left({\hat{h}}^{\mathrm{re}},{\hat{h}}^{\mathrm{im}}\right)$ of unsigned integers. Moreover, the quantizer delivers a pair $\left(y_{k}^{\mathrm{re}},y_{k}^{\mathrm{im}}\right)$ for each symbol $s_{k}$. The task of an information bottleneck demodulator equivalent for the considered channel is extracting relevant information on $s_{k}$ from $\left({\hat{h}}^{\mathrm{re}},{\hat{h}}^{\mathrm{im}}\right)$ and $\left(y_{k}^{\mathrm{re}},y_{k}^{\mathrm{im}}\right)$.

**Figure 8:**
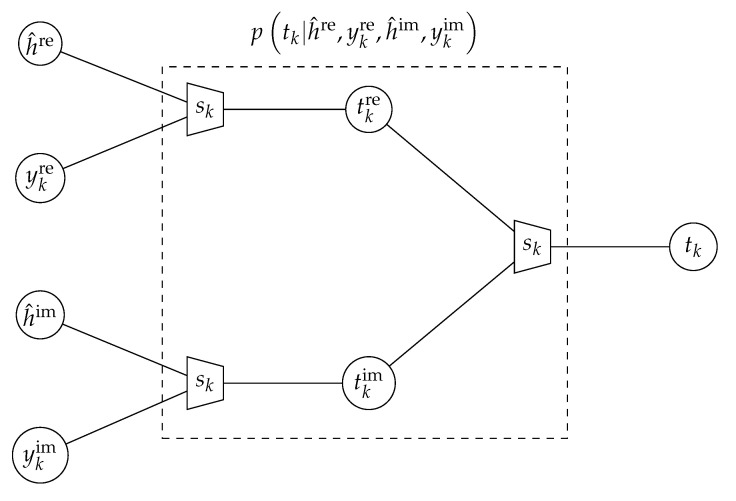
Information bottleneck graph of the detection scheme for the information bottleneck receiver. The detection scheme first extracts information on $s_{k}$ from $\left(y_{k}^{\mathrm{re}},{\hat{h}}^{\mathrm{re}}\right)$ and $\left(y_{k}^{\mathrm{im}},{\hat{h}}^{\mathrm{im}}\right)$ independently. Afterwards, it yields a an integer output $t_{k}$ from $t_{k}^{\mathrm{re}}$ and $t_{k}^{\mathrm{im}}$ that is informative about $s_{k}$ using the mapping $p(t_{k}|t_{k}^{\mathrm{re}},t_{k}^{\mathrm{im}})$.

Figure 8 shows the information bottleneck graph performing this task. In this graph, one first successively combines ${\hat{h}}^{\mathrm{re}},y_{k}^{\mathrm{re}}$ and ${\hat{h}}^{\mathrm{im}},y_{k}^{\mathrm{im}}$ to obtain $t_{k}^{\mathrm{re}}$ and $t_{k}^{\mathrm{im}}$. The compression mappings $p\left(t_{k}^{\mathrm{re}}|{\hat{h}}^{\mathrm{re}},y_{k}^{\mathrm{re}}\right)$ and $p\left(t_{k}^{\mathrm{im}}|{\hat{h}}^{\mathrm{im}},y_{k}^{\mathrm{im}}\right)$ are identical, as their inputs are identically distributed. To design $p\left(t_{k}^{\mathrm{re}}|{\hat{h}}^{\mathrm{re}},y_{k}^{\mathrm{re}}\right)$ with an information bottleneck algorithm, one needs the joint probability distribution

(17) $$p\left(s_{k},{\hat{h}}^{\mathrm{re}},y_{k}^{\mathrm{re}}\right)=\sum_{h^{\mathrm{re}}=0}^{2^{q}-1}p\left(h^{\mathrm{re}},y_{k}^{\mathrm{re}}|s_{k}\right)\underset{p\left({\hat{h}}^{\mathrm{re}}|h^{\mathrm{re}}\right)}{\underset{︸}{\frac{p\left(h^{\mathrm{re}},{\hat{h}}^{\mathrm{re}}\right)}{p\left(h^{\mathrm{re}}\right)}}}p\left(s_{k}\right).$$

In Equation (17), the distributions $p\left(h^{\mathrm{re}},{\hat{h}}^{\mathrm{re}}\right)$ and $p\left(h^{\mathrm{re}}\right)$ are known from the design of the channel estimation lookup table. The distribution $p\left(h^{\mathrm{re}},y_{k}^{\mathrm{re}}|s_{k}\right)$ is again an integral of a multivariate Gaussian distribution, where

(18) $$p\left(h^{\mathrm{re}},y_{k}^{\mathrm{re}}\right|s_{k})=\int_{-\infty }^{+\infty }\int_{-\infty }^{+\infty }p\left({\tilde{h}}^{\mathrm{re}},{\tilde{y}}_{k}^{\mathrm{re}}|s_{k}\right)p\left(h^{\mathrm{re}}|{\tilde{h}}^{\mathrm{re}}\right)p\left(y_{k}^{\mathrm{re}}|{\tilde{y}}_{k}^{\mathrm{re}}\right)\;d\;{\tilde{h}}^{\mathrm{re}}\;d\;{\tilde{y}}_{k}^{\mathrm{re}}.$$

Again, the integral can be solved using the method from [62]. The required covariance matrix is obtained analogously with Equation (14).

An important note is that the design of the final concatenated information bottleneck compression mapping $p(t_{k}|t_{k}^{\mathrm{re}},t_{k}^{\mathrm{im}})$ inherently delivers the distributions $p(s_{k}|t_{k})$ and $p(t_{k})$ and, therefore, $p(s_{k},t_{k})$. Reviewing Figure 4, the concatenation of the transmitter, channel, quantizer and the proposed concatenation of information bottleneck compression mappings therefore corresponds to a discrete input, discrete output transmission scheme. This discrete input, discrete output transmission scheme delivers $t_{k}\in \left\{0,1,\ldots ,2^{q^{\det}}-1\right\}$ for each transmitted modulation symbol $s_{k}$ at the receiving end, where $2^{q^{\det}}$ is the output cardinality of the information bottleneck algorithm used to design $p(t_{k}|t_{k}^{\mathrm{re}},t_{k}^{\mathrm{im}})$. The statistical properties of $\left(s_{k},t_{k}\right)$ and the preserved relevant mutual information I$\left(S_{k};T_{k}\right)$ are completely determined by $p(s_{k},t_{k})$, which describes a discrete source and channel model for the considered data transmission scheme. This model is needed to construct the iterative information bottleneck LDPC decoder from Figure 6. The design of this LDPC decoder is sketched in the following section. More details can be found in [18,61].

#### 2.3.3. Information Bottleneck LDPC Decoder Design

As briefly mentioned above, the design of an information bottleneck LDPC decoder requires knowledge of the joint probability distribution $p(s_{k},t_{k})$. Luckily, this distribution is delivered inherently by the information bottleneck algorithm used to design the detection scheme from the previous section.

This probability distribution is iteratively processed in a discretized density evolution algorithm [12,18,61] to construct a coarsely quantized information bottleneck LDPC decoder. The density evolution algorithm is needed to determine the input distributions of the information bottleneck algorithms used to design the lookup tables that implement the variable and the check node operations of a message passing LDPC decoder in the subsequent decoder iterations. The lookup tables only process and deliver integer indices and therefore are fundamentally different in their operation than conventional LDPC decoders, which process real-valued LLRs.

**Figure 9:**
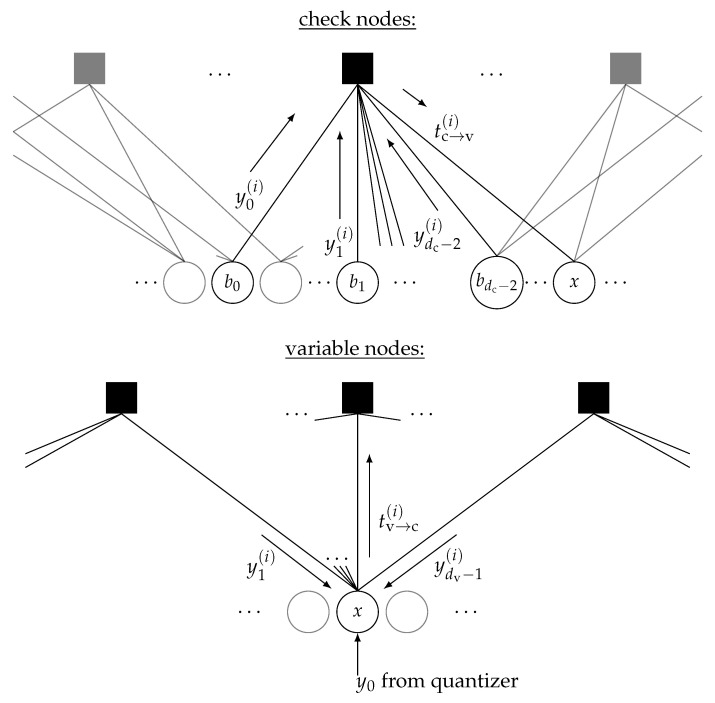
Message generation of a check node and a variable node in an iterative information bottleneck LDPC decoder. The nodes generate integer messages for their connected edges.

Figure 9 gives an overview of the notation used to describe the design of the information bottleneck LDPC decoder. We consider a regular LDPC code with a variable node degree $d_{v}$ and check node degree $d_{c}$. In decoder iteration *i*, the variable nodes pass quantized messages (i.e., unsigned integers $y_{k}^{\left(i\right)}$) to the check nodes. The check nodes generate outgoing integer messages $t_{c\to v}^{\left(i\right)}$ for all their connected edges from these incoming messages. This process is illustrated for one particular target edge at the top of Figure 9. The check node operation is designed such that the mutual information shared between the outgoing message and the codeword bit that this message represents in the LDPC codeword is maximized with the information bottleneck method. This codeword bit is denoted by *x* in the upper part of Figure 9. The other $d_{c}-1$ codeword bits connected to the check node are denoted by $b_{0},b_{1},\ldots ,b_{d_{c}-2}$.

As the variable nodes pass the received messages from the channel to the check nodes in the first decoding iteration, in principle, the check node operation could be designed in a straightforward manner by feeding the joint probability distribution

(19) $$p\left(x,y_{0}^{\left(i\right)},y_{1}^{\left(i\right)},\ldots ,y_{d_{c}-2}^{\left(i\right)}\right)=\sum_{\begin{array}{c} (b_{0},b_{1},\ldots ,b_{d_{c}-2}):\\ x=b_{0}⊕b_{1}⊕\ldots ⊕b_{d_{c}-2} \end{array}}\prod_{k=0}^{d_{c}-2}p\left(b_{k},y_{k}\right)$$

to an information bottleneck algorithm. This approach, however, suffers from intractable complexity, as the number of input configurations $\left(y_{0}^{\left(i\right)},y_{1}^{\left(i\right)},\ldots ,y_{d_{c}-2}^{\left(i\right)}\right)$ grows exponentially with the node degree $d_{c}$. As a result, the authors of [12] proposed splitting the check node operation into a series of concatenated two-input operations, exactly as was performed in Section 2.3.1 for the channel estimation scheme to reduce the complexity. In this way, a series of concatenated two-input lookup tables can be designed with an information bottleneck algorithm that processes unsigned integers and aims to maximize the preserved relevant information I$\left(X;T_{c\to v}^{\left(i\right)}\right)$.

The information bottleneck algorithm used for the design of the check node operation finally delivers $p(x,t_{c\to v}^{\left(i\right)})$. Based on this distribution, a very similar process can be used to design the variable node operation depicted in the bottom part of Figure 9. The variable nodes create outgoing messages $t_{v\to c}^{\left(i\right)}$ that shall be highly informative of their corresponding codeword bit *x* (i.e., I$(X;T_{v\to c}^{\left(i\right)})\to \max$). To obtain extrinsic information on the codeword bit *x*, the variable nodes process $d_{v}-1$ messages from the connected check nodes and a message $y_{0}$ from the channel. In the considered data transmission scheme, the channel messages correspond to $t_{k}\in \left\{0,1,\ldots ,2^{q^{\mathrm{ce}}}-1\right\}$ from the detection scheme described in Section 2.3.2. As a result, the design of the variable node operation with the information bottleneck method processes the joint probability distribution $p(x,y_{0})$, which is equivalent to $p(s_{k},t_{k})$ and $p(x,t_{c\to v}^{\left(i\right)})$ from the design process of the check node operation. For the equations needed to obtain all involved joint probability distributions, the reader is asked to refer to [18,61]. Most importantly, the information bottleneck algorithm applied to the variable node design delivers $p(x,t_{v\to c}^{\left(i\right)})$. This distribution has to be processed to design the check node operation for the next decoder iteration i+1, which will in turn deliver $p(x,t_{c\to v}^{\left(i+1\right)})$ for the check nodes in the next decoding iteration. This naturally suggests an iterative algorithm to determine lookup tables used as node operations in an information bottleneck LDPC decoder. This algorithm is iterated for a desired number of $i_{\max}$ iterations of the constructed LDPC decoder, and the lookup tables constructed for the variable and the check node operations in each iteration of the decoder are stored.

Finally, an important note on the proposed receiver design shall be made. Obviously, the joint probability distributions that are processed for the decoder design and also for the channel estimation and detection scheme studied before depend on the channel conditions. As a result, the reader might expect that the lookup tables designed with the information bottleneck method need to be adjusted to the signal-to-noise ratio or, equivalently, to the current $E_{b}/N_{0}$ on the channel. As the design process of the detection, channel estimation and especially the LDPC decoder is computationally quite complex and involves numerous information bottleneck algorithms, this would clearly question the practical use of the proposed receiver design. However, interestingly, the proposed receiver design provides excellent performance, even with information bottleneck lookup tables that are used being mismatched to the actual channel conditions. This allows conducting the entire receiver design process offline to store the resulting lookup tables and to use them in a receiver with coarse quantization and very simple operations only. We will analyze the performance of the resulting receiver and compare it to several reference receivers in the following to prove our statement on excellent performance.

#### 2.3.4. Comparison of Iterative Receiver Performances

In this section, we investigate the bit error rate performance of the proposed information bottleneck receiver and compare it to the one of several reference receivers. As mentioned above, the proposed information bottleneck receiver from Figure 6 was designed for a manually chosen design $E_{b}/N_{0}$ and was not matched to the actual $E_{b}/N_{0}$ on the channel in the results presented next. The lookup tables were constructed offline with the information bottleneck method.

**Figure 10:**
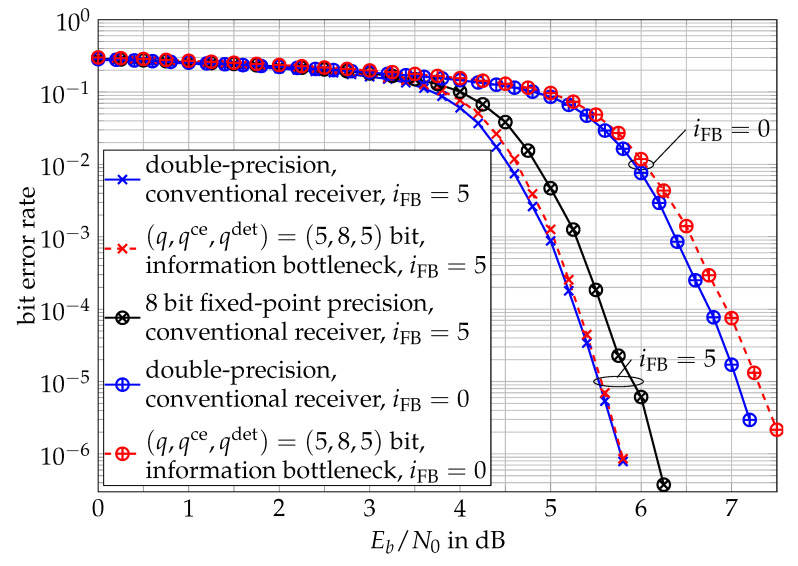
Bit error rates of different quantized receivers and the conventional receiver from Figure 6, which did not suffer from a quantization loss at all. The quantized information bottleneck receiver with q=5 bit channel output quantization, $q^{\mathrm{ce}}=8$ bit channel estimation and $q^{\det}=5$ bit detection and LDPC decoding met the performance of the double-precision reference receiver for $i_{\mathrm{FB}}=5$ feedback iterations.

The bit error rate performances of the considered receivers are compared in Figure 10 for a number of $i_{\mathrm{FB}}=0$ and $i_{\mathrm{FB}}=5$ feedback iterations of the decision feedback channel estimation. The applied LDPC code was a length of 8000 $\left(d_{v},d_{c}\right)=\left(3,6\right)$ regular LDPC code from [63]. In the studied scenario, we considered a block fading channel that was constant for B=35 symbol durations before a new independent channel coefficient was drawn, which weighted the transmitted symbols according to Equation (9). The first P=3 symbols transmitted for each of these blocks were the known pilot symbols for the initial channel estimation.

The quantization bit widths of the information bottleneck receiver were chosen as follows. We used q=5 bits per sample for channel output quantization in the real and imaginary parts of the received signal, respectively, $q^{\mathrm{ce}}=8$ bits for channel estimation and $q^{\det}=5$ bits for the detection lookup table.

We also used $q^{\det}=5$ bit messages in the information bottleneck LDPC decoder. The decoder applied $i_{\max}=25$ iterations in each of the outer feedback iterations for the channel estimation. In each outer iteration, the $N_{\mathrm{FB}}=20$ most reliable bit decisions from this decoder in each block were fed to the feedback channel estimator illustrated in Figure 7 to supplement the channel estimation. The red curves in Figure 10 show the bit error rate performance of the proposed information bottleneck receiver with these parameters for $i_{\mathrm{FB}}=0$ and $i_{\mathrm{FB}}=5$ outer iterations of the decision feedback loop for channel estimation. A comparison of the receiver’s performance for $i_{\mathrm{FB}}=0$ and $i_{\mathrm{FB}}=5$ outer iterations of the channel estimation feedback loop clearly illustrates the gain resulting from improving the initial channel estimate based on the decision feedback from the channel decoder.

The blue curves in Figure 10 correspond to the conventional receiver chain from Figure 6 with the respective numbers of feedback iterations $i_{\mathrm{FB}}=0$ and $i_{\mathrm{FB}}=5$ and also used $i_{\mathrm{FB}}=25$ iterations of the conventional belief propagation LDPC decoder. We want to stress again that this receiver does not suffer from any mentionable quantization loss.

Despite that, it can clearly be observed that the performance of the quantized information bottleneck receiver chain that only processed unsigned integers and replaced all conventional arithmetical operations in the signal processing algorithms with simple lookup operations for $i_{\mathrm{FB}}=5$ iterations was tremendously close to the one of the conventional reference receiver. It is also observable that without feedback iterations (i.e., $i_{\mathrm{FB}}=0$), the loss of the information bottleneck receiver was slightly higher. The reason for this is that all of the lookup tables in the information bottleneck receiver were designed only for a single design $E_{b}/N_{0}$, which we optimized to yield the best performance for the case with $i_{\mathrm{FB}}=5$ feedback iterations. It is, however, possible to also tune the design $E_{b}/N_{0}$ to minimize the performance gap with respect to the non-quantized receiver without feedback iterations to achieve a negligible loss over $E_{b}/N_{0}$ for $i_{\mathrm{FB}}=0$ [61].

The numbers of bits $\left(q,q^{\mathrm{ce}},q^{\det}\right)=\left(5,8,5\right)$ used in the information bottleneck signal processing units were determined by carefully analyzing which settings resulted in the optimum performance. While for the detection and decoding stage we found $q^{\det}=5$ bit processing sufficient, during our investigations, we noted that the channel estimation appeared to require a larger bit width of $q^{\mathrm{ce}}=8$ bits to perform that close to the non-quantized reference system. A possible intuitive explanation is that other than for all the detection and decoding parts of the receiver, the relevant variable of the channel estimator $H^{\mathrm{re}}=f_{Q}\left({\tilde{H}}^{\mathrm{re}}\right)$ is not binary. Instead, with a *q*-bit channel output quantizer $f_{Q}\left(.\right)$ in place, it can take $2^{q}$ different values. This intuitively suggests that in order to achieve close-to-optimum performance, the channel estimation stage requires a larger bit width to preserve the relevant information.

As another reference, the bit error rate of an 8-bit fixed-point implementation of the conventional receiver is included in Figure 10 for $i_{\mathrm{FB}}=5$ feedback iterations in black. This receiver used the Q5.3 fixed-point format in the detection and channel estimation stages and the Q4.4 fixed-point format for the messages in the belief propagation decoder. In this notation, Qm.n denotes using *m* bits for the integer part and *n* bits for the fractional part of the processed fixed-point numbers. In order to not disadvantage the conventional receiver chain, all other possible combinations of 8-bit fixed-point formats were also tested, but the chosen selection offered the best performance. The 8-bit fixed-point conventional receiver was clearly outperformed by the information bottleneck receiver. This is particularly interesting because the information bottleneck receiver only used 8-bit integers in its channel estimation stage and shorter 5-bit integers in all other detection and decoding stages.

So far, Figure 10 shows that the performance of the proposed receiver was very close to that of the conventional receiver with double-precision if $i_{\mathrm{FB}}=5$ feedback iterations were performed. It is, however, still unclear whether or not the same information bottleneck receiver suffers from performance degradation if fewer feedback iterations are performed.

**Figure 11:**
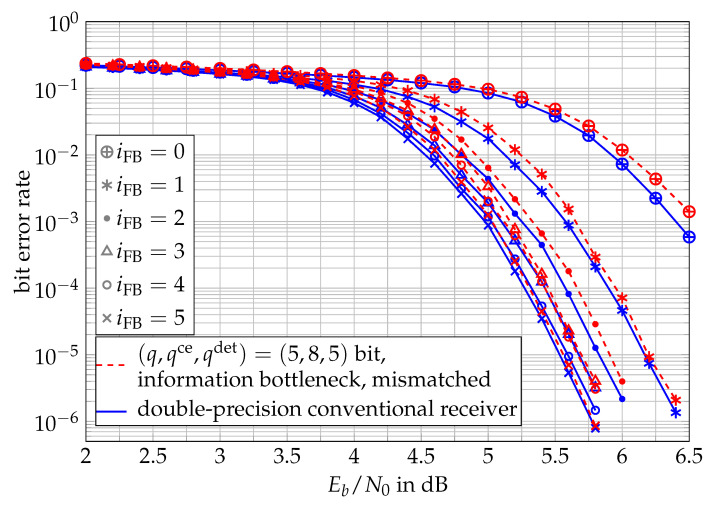
Bit error rates of different receiver implementations as a function of the number $i_{\mathrm{FB}}$ of feedback iterations. The quantized information bottleneck receiver with q=5-bit channel output quantization, $q^{\mathrm{ce}}=8$-bit channel estimation and $q^{\det}=5$-bit detection and LDPC decoding offered performance similar to the double-precision conventional receiver for all investigated $i_{\mathrm{FB}}$.

As a result, Figure 11 shows the bit error rates of both receivers for all $i_{\mathrm{FB}}\in \left\{0,1,2,3,4,5\right\}$. The figure illustrates that the performance of the information bottleneck receiver can compete with the double-precision conventional receiver for all investigated numbers of feedback iterations $i_{\mathrm{FB}}$. The figure also indicates that the gains of the feedback loop were very significant in the first three feedback iterations for both receivers and then became less significant. It was found that using more than five feedback iterations hardly improved the bit error rate performance of any investigated receiver.

As the most important result so far, we can summarize that the information bottleneck design of the proposed receiver yielded a quantized information bottleneck receiver with very simple and homogenous operations that could deliver performance practically identical to that of a conventional receiver with double-precision arithmetic. This receiver employs advanced signal processing concepts, such as decision feedback for quantized channel estimation and quantized LDPC decoding. All of the signal processing operations used in the receiver were designed using the same principle of maximizing the flow of relevant information through the quantized signal processing operations of the receiver with the information bottleneck method.

## 3. Parameter Learning of Trainable Functions to Maximize the Relevant Information

In the information bottleneck receiver presented above, as we have often emphasized, all signal processing operations were implemented as lookup tables. As mentioned in the introduction, however, there exist several other approaches to implementing information bottleneck signal processing units in the literature, especially in the context of LDPC decoding. In this section, our goal is to provide an overview of the lookup table approach, the computational domain methods used in [24,25,30,31] and the neural network approach from [28,29]. All can be seen as mappings $t=f_{\theta }\left(y\right)$ implied by a trainable function $f_{\theta }\left(y\right)$, with parameters θ that are trained to maximize the preserved relevant information I(X;T).

**Figure 12:**
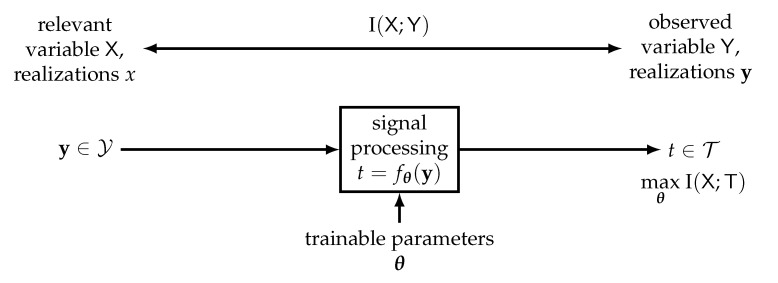
General illustration of an information bottleneck signal processing unit. The signal processing unit consists of a trainable function with parameters θ that can be learned to maximize I(X;T).

This view on information bottleneck signal processing is depicted in Figure 12 and was introduced previously in [64,65].

### 3.1. Lookup Tables

As mentioned in Section 2.1, it is intuitive that any deterministic compression mapping p(t|y) for discrete *t* and *y* can be implemented in a lookup table. For that purpose, one just has to store the respective t∈T for all possible y∈Y. Of course, this also holds if the realizations of Y are random vectors $y=[y_{0},y_{1},\ldots ,y_{N-1}]$.

For simplicity, we assume that $y_{n}\in {\left\{0,1,\ldots ,|Y\right|}_{n}-1\}$ in this section (i.e., the $y_{n}$ in vector y are unsigned integers). Please note that the elements of the event space are totally irrelevant for the mutual information I(X;Y), as this mutual information only depends on the probabilities implied by p(x,y),p(x) and p(y). We can easily understand a lookup table holding the *t* for each possible y as a trainable function $f_{\theta }\left(y\right)$ with parameters θ.

Consider a software implementation of a lookup table consisting of a row vector $\theta =[\theta_{0},\theta_{1},\ldots ,\theta_{|Y|-1}]$. This vector holds the outcome *t* for every possible input vector y at a certain position that is termed the address. To obtain the output *t*, the input y has to be mapped onto the address of the corresponding output *t* in the vector. Let the function implementing this address transformation be denoted by ξ(y). It is not important which address transformation ξ(y) is used as long as it maps the input vector y onto the address of the corresponding t∈T uniquely. Then, the lookup table delivers $t=f_{\theta }\left(y\right)=\theta_{\xi (y)}$ by just accessing the vector θ at the calculated address.

With this quite formal description of a simple lookup table, it is clear that the problem of maximizing the preserved relevant information I(X;T) with t∈T and y∈Y is formally equivalent to determining a vector of the optimum discrete parameters:

(20) $$\theta_{\beta \to \infty }^{\mathrm{IB}}=\arg\max_{\theta }I\left(X;f_{\theta }\left(Y\right)\right).$$

Therefore, we can summarize that any deterministic mapping of y onto *t* that is implemented in a lookup table and determined to maximize the preserved relevant information with an information bottleneck algorithm can equivalently be interpreted as a trainable function $f_{\theta }\left(y\right)$ with a maximum of |Y| discrete parameters $\theta_{\xi (y)}\in T$. The number of parameters |Y| is identical to all possible input configurations y∈Y. This allows representing arbitrary mappings in this setup and has the consequence that a lookup table can implement every possible input/output relation for discrete y and *t*, including one that maximizes I(X;T) globally. This holds independent of the probability distribution p(x,y). Yet, we note that finding an optimum mapping with the available information bottleneck algorithms is not guaranteed. However, for discrete y∈Y and discrete t∈T, we can conclude that no other mapping can offer more flexibility than a lookup table. Therefore, in theory, a lookup table can be seen as an optimum choice in terms of providing the possibility to maximize I$\left(X;f_{\theta }\left(Y\right)\right)$ with properly tuned parameters θ. The way to learn these parameters in the receiver presented above was feeding the joint probability distributions p(x,y) to the information bottleneck algorithms.

Unfortunately, however, problems arise if the number *N* of scalar inputs to be processed by a lookup table is large, as was the case, for example, for the node operations in the information bottleneck LDPC decoders from Section 2.3.3. These operations have to process a huge number of scalar input variables, and the number of possible input configurations of a lookup table scales exponentially with the number of scalar inputs. However, we have seen that in the studied cases, it was possible to split the signal processing operations designed as information bottleneck lookup tables into concatenated two-input tables to reduce the complexity. This, however, results in a concatenation of several lossy two-input lookup tables. Hence, the authors of [24,25] argued reasonably that such a splitting procedure likely results in losses of the preserved relevant information in comparison with an input/output mapping that can process all scalar inputs consolidated in the input vector y at once.

### 3.2. Computational Domain Technique

**Figure 13:**
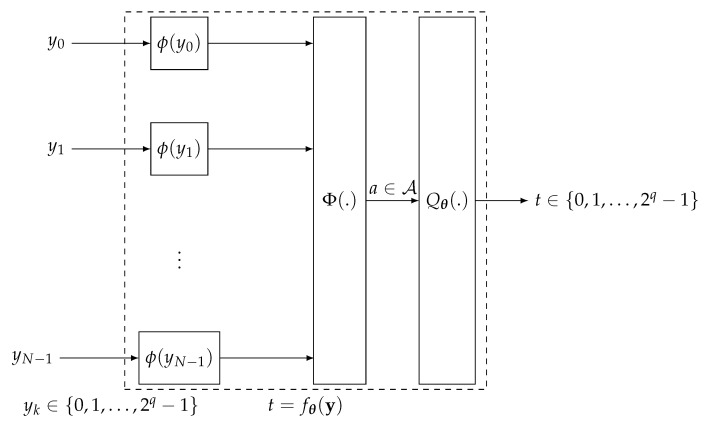
Illustration of the computational domain approach for information bottleneck-like LDPC decoding from [24,25]. Figure adapted from [24,25] with minor adaptions of the notation.

The authors of [24,25] proposed an interesting idea to implement check and variable node operations of LDPC decoders that, just as with the information bottleneck approaches based on lookup tables described above, aims at maximizing the preserved relevant information under compression. Their approach is called mutual information-maximizing quantized belief propagation decoding. We briefly recall this approach that is illustrated in Figure 13 in the following.

The authors of [24,25] distinguished between variable and check node operations in an LDPC decoder. Here, however, we study a general input/output relation that maps an incoming vector $y=[y_{0},y_{1},\ldots ,y_{N-1}]$ onto an outgoing *t*. For simplicity, we assume the most practical approach of all $y_{n}$ and *t* to be from the same set $\left\{0,1,\ldots ,2^{q}-1\right\}$ of *q*-bit unsigned integers. The computational domain approach consists of three subsequent steps [24,25]:

1. Use a predefined reconstruction function ϕ(.) to transfer the incoming messages $y_{n}$ to numbers $\phi (y_{n})$ in a computational domain D;
2. Use a function $\Phi :D^{N}⟶A$ to process the numbers in the computational domain and to map them onto a single number a∈A;
3. Apply a scalar quantizer $Q_{\theta }\left(.\right)$ with $2^{q}-1$ ordered thresholds $\theta =[\theta_{0},\theta_{1},\ldots ,\theta_{2^{q}-2}]$ on *a* that quantizes a∈A back to the set $\left\{0,1,\ldots ,2^{q}-1\right\}$.

In [24,25], certain reconstruction functions for the check and variable nodes of LDPC decoders and also reasonable functions Φ(.) for these applications, which we do not recall here in detail for brevity, are proposed.

The idea of maximizing the preserved relevant mutual information in the information bottleneck sense is applied in the design of the scalar quantizer $Q_{\theta }\left(.\right)$ in the computational domain approach from [24,25]. The thresholds $\theta =[\theta_{0},\theta_{1},\ldots ,\theta_{2^{q}-2}]$ of this quantizer are chosen such that I(X;T) is maximized.

Of course, one can understand the quantization thresholds stored in vector $\theta =[\theta_{0},\theta_{1},\ldots ,\theta_{2^{q}-2}]$ as parameters of a function implementing the node operation

(21) $$t=f_{\theta }\left(y\right)=Q_{\theta }\left(\Phi \left(\phi \left(y_{0}\right),\phi \left(y_{1}\right),\ldots ,\phi \left(y_{N-1}\right)\right)\right),$$

such that the design of the node operation ends up in the exact same problem formulation as that given in Equation (20) (that is, the learning parameters θ of a trainable function $f_{\theta }\left(y\right)$ such that I$(X;f_{\theta }\left(Y\right))⟶\max$).

The major difference is that the number of parameters that are tuned to maximize I$\left(X;f_{\theta }\left(Y\right)\right)$ can be drastically reduced in the computational domain approach with respect to the lookup table. Essentially, only $2^{q}-1$ quantization thresholds instead of $2^{qN}$ entries of a lookup table need to be learned and optimized to maximize I(X;T). Interestingly, the computational domain approach employs arithmetical operations in the function Φ(.). In return, it needs much fewer parameters to preserve significant amounts of I(X;T) than the lookup table approach. We note, however, that the choice of the reconstruction function ϕ(.) and the function Φ(.) as well as their abilities to preserve significant amounts of I(X;T) are problem-specific and depend on p(x,y).

It has to be appreciated that the proposed method from [24,25] offers excellent bit error rate performance for LDPC decoding.

**Figure 14:**
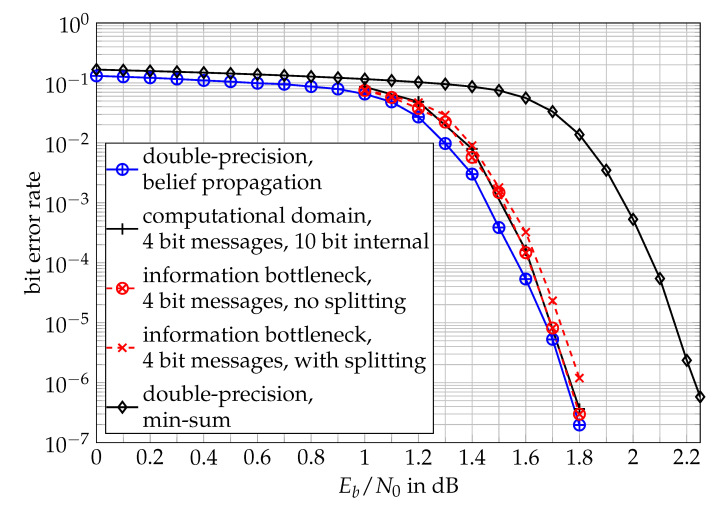
Bit error rates of several LDPC decoders for data transmission with BPSK over an additive white Gaussian noise channel. The applied code was a (3,6) regular LDPC code. All decoders conducted $i_{\max}=50$ decoding iterations. The computational domain approach refers to [24].

To illustrate this fact, Figure 14 compares the bit error rate performances of the computational domain approach and two lookup table-based information bottleneck decoders for the $\left(d_{v},d_{c}\right)=\left(3,6\right)$ regular LDPC code that was already used in Section 2.3.4. For these simulations, all quantized decoders exchanged q=4-bit messages in the iterative message passing process for their respective decoding. The bit width used internally to represent a∈A in the computational domain decoders was 10 bits for the check nodes and variable nodes. The shown results for the computational domain decoder were adopted from [24]. The bit error rate curves refer to data transmission with BPSK modulation over an additive white Gaussian noise channel. The number of decoder iterations was $i_{\max}=50$ for all investigated decoders.

The relatively small node degrees of the applied code allow constructing lookup table-based information bottleneck decoders with and without splitting the node operation into two-input operations. We want to mention that the decoder without splitting has an impractically large memory demand but shall be used as a benchmark here. For the relatively small node degrees of the (3,6) regular LDPC code used here, the lookup tables in the decoder without splitting already had more than 300 million entries [28].

As can be seen, the computational domain decoder performed just as well as a lookup table-based decoder without internal splitting of the node operations. Both quantized decoders effectively reached the performance of the non-quantized belief propagation decoder with double-precision up to a negligible gap over $E_{b}/N_{0}$.

As can also be seen, splitting the node operation of the information bottleneck decoder into a series of two-input operations offered slightly worse performance but yielded much smaller lookup tables in return. The number of lookup table entries used for this decoder was just 384,000 and therefore drastically reduced to a tractable number in comparison with the lookup table-based decoder without two-input splitting of the node operations.

Again, we want to stress here that the information bottleneck decoders and the computational domain decoder [24] were entirely constructed offline and used mismatched to the $E_{b}/N_{0}$ on the channel. For reference, the bit error rate of the well-known min-sum decoder with double-precision is also shown in Figure 14. This decoder was outperformed by all decoders designed with the information bottleneck principle of maximizing the preserved relevant information.

Finally, we note that the computational domain approach is well suited for LDPC decoding. Thus far, to the best of our knowledge, studies of computational domain approaches that maximize the relevant information for other applications of information bottleneck signal processing such as the channel estimation studied in Section 2.3.1, cannot be found in the literature.

### 3.3. Neural Networks

Another idea to implement information bottleneck signal processing for LDPC decoding was introduced in [28]. In order to get rid of the need for implementing very large lookup tables to process the incoming messages of a check or variable node in one shot (i.e., without splitting the node operation), neural networks were trained to maximize the preserved relevant information. Learning the parameters of such neural networks was conducted in a supervised manner in [28] first. To accomplish this, as a first step, lookup tables that did not use two-input table splitting were constructed with the information bottleneck method for the variable and check node operations. Afterwards, different neural network structures were trained to mimic the resulting node operations. The key is, of course, that the trained networks have much fewer parameters θ than there are entries in the original lookup tables.

We want to recall a certain neural network structure proposed to implement the node operations in [28] here. This neural network directly inputs the binary representations of the integer messages exchanged to implement the node operations of the check or the variable nodes. Moreover, this network is iteration-aware [28], meaning that the decoder iteration *i* is used as an additional input to the network together with the incoming messages of a node. In this way, only one network for the check nodes and one network for the variable nodes are required to implement all node operations for all iterations of the decoder.

For the (3,6) regular LDPC code considered in the prior section, this architecture results in a neural network-based decoder with 25,564 parameters of the involved neural networks. Recall again that the lookup table-based information bottleneck decoder without split node operations required more than 300 million lookup table entries, and the one with splitting required 384,000 lookup table entries (for more quantitative details, see Table I from [28]).

**Figure 15:**
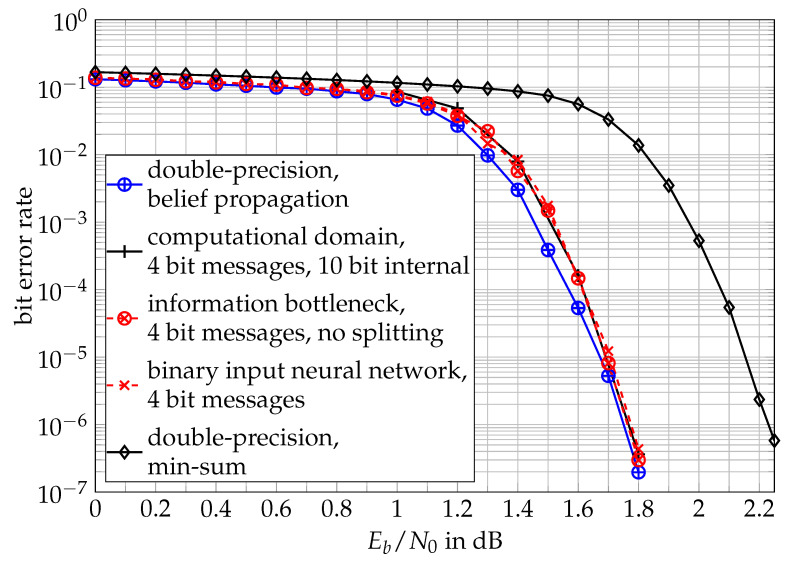
Bit error rates of several LDPC decoders for data transmission with BPSK over an additive white Gaussian noise channel. The applied code was a (3,6) regular LDPC code. All decoders conducted $i_{\max}=50$ decoding iterations. The computational domain approach refers to [24].

Figure 15 shows the bit error rate results of the neural network method for implementation of the node operations from [28]. The simulation setup, the used LDPC code and the number of decoding iterations were the same as those for the computational domain approach from the prior section.

As can clearly be seen, the neural network implementation of the node operations achieved a practically identical performance to the lookup table-based information bottleneck decoder without split node operations as well as the computational domain decoder studied before. All these decoders approach the performance of the double-precision belief propagation decoder with quite different approaches to implement the node operations which, however, all are motivated by the information bottleneck idea of maximizing the preserved relevant information under quantization.

The supervised learning approach to train a neural network that shall maximize I(X;T) presented in [28] still requires designing huge lookup tables used to generate the training data as an intermediate step. Hence, the idea was also extended to unsupervised learning in [29] such that the parameters of the neural networks involved were directly trained to maximize the preserved relevant information with standard gradient methods that are commonly used to train the parameters of neural networks. This eliminates the intermediate step of designing lookup tables with impractical sizes for decoder implementation. Moreover, it directly leads back to the problem formulation from Equation (20), which again illustrates that this neural network-based approach of information bottleneck signal processing can also be understood as the learning parameters of a trainable function that shall maximize the preserved relevant information.

However, if the number *N* of scalar inputs consolidated in the input vector y is large, representing p(x,y) in closed form to calculate p(x,t) and from that I(X;T) is also infeasible. As a way out, a number of samples (x,y) can be used to determine the corresponding $t=f_{\theta }\left(y\right)$ for all samples and, from that, estimate p(x,t) and I(X;T).

Finally, we want to mention that obtaining an accurate estimate of I(X;T) in this unsupervised learning procedure typically requires a vast amount of samples (x,y) if the number of scalar inputs consolidated in y is large. This problem is well known in the machine learning community and often referred to as the curse of dimensionality. However, this number of samples only influences the construction complexity of the node operations in the LDPC decoder but not their implementation complexity.

### 3.4. Further Discussion, Other Approaches and Future Work

The discussion of the lookup table approach, the computational domain approach and the neural network approach to information bottleneck signal processing reveals the key principles of information bottleneck signal processing. These are choosing appropriate functions $f_{\theta }\left(y\right)$ with trainable parameters θ and determining the values of these parameters that maximize the relevant information. The functions used should only use a small number of simple arithmetical operations, and the number of parameters should also be small for complexity reasons.

As was discussed above, lookup tables have strengths regarding flexibility, as for discrete inputs y and discrete outputs *t*, they can implement arbitrary input/output relations $t=f_{\theta }\left(y\right)$. However, they often need too many parameters if the number of inputs is large. The studied computational domain approach requires much fewer parameters but needs a sophisticated choice for the reconstruction function ϕ(.) and the function Φ(.) for the signal processing problem of interest, (e.g., LDPC decoding). Neural network approaches offer an almost endless choice of network architectures. These are determined by the number of layers, the types of the layers and their respective activation functions. All these aspects influence their implementation complexity and the number of parameters. However, neural networks enable realizing almost arbitrary mappings with proper architectures and a reasonable number of parameters. Moreover, they can be trained efficiently using gradient-based algorithms.

In future work, it will be interesting to further study flexible, trainable functions for information bottleneck signal processing units. To illustrate that many more options exist, we briefly mention a quite different approach from [64,65] as an example. This approach is based on efficient nearest neighbor search algorithms in graphs. These search algorithms are used to implement trainable functions $f_{\theta }\left(y\right)$ that aim to maximize I(X;T) in [64,65].

**Figure 16:**
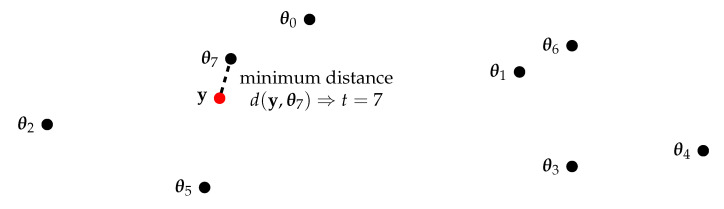
Illustration of a mapping $t=f_{\theta }\left(y\right)$ based on the nearest neighbor search. The output *t* is the index of the nearest neighbor $\theta_{t}$ of y. It can be found by using graph-based algorithms efficiently.

The mappings $t=f_{\theta }\left(y\right)$ in [64,65] use a small number of distance calculations between the incoming vector y and some trained parameter vectors $\theta_{t}$ to determine the system output *t* such that I(X;T)→max. An exemplary nearest neighbor search is illustrated in Figure 16.

In the figure, the parameters $\theta =[\theta_{0},\theta_{1},\ldots ,\theta_{7}]$ reflect the positions of the points labeled $\theta_{t}$ in the two-dimensional plain. In addition, an exemplary two-dimensional input vector y is shown as a red dot. The outcome of the operation $t=f_{\theta }\left(y\right)$ is the integer index *t* of an approximate or exact nearest neighbor $\theta_{t}$ of the input vector y under some arbitrary distance measure $d(y,\theta_{t})$. In Figure 16, the distance is the Euclidean distance, and the exact nearest neighbor is considered for illustration purposes. Please note that it is not required to calculate all possible distances $d(y,\theta_{t})$ to determine the system output *t*. Instead, either an exact or approximate nearest neighbor search using very efficient search algorithms in graph structures of the parameter vectors $\theta_{t}$ can be applied [64,65]. The parameters involved in nearest neighbor search-based mappings were trained using genetic algorithms in [64,65]. Genetic algorithms are parameter optimization algorithms which are inspired by the natural evolution of the species [66].

Clearly, this realization of a mapping $t=f_{\theta }\left(y\right)$, which aims to maximize I(X;T), differs a lot from the lookup table-based method, the computational domain approach and also the neural network method studied above. Yet, the results from [64,65] prove it to be quite powerful in terms of the preservation of I(X;T), with few parameters for distance-based quantization and demodulation in communication receivers. This illustrates that there is a lot of potential in finding different flexible and simple trainable functions $f_{\theta }\left(y\right)$ to design information bottleneck signal processing units for different signal processing problems in the future. In addition, efficient training of the parameters involved offers interesting research directions.

## 4. Conclusions

In this article, we first gave an overview of the information bottleneck method and explained how it can be linked to the fundamental task of a digital communication receiver. The application of the information bottleneck method for receiver-side signal processing in this context effectively allows building quantized signal processing units that aim to maximize the relevant information that flows through them. This concept is fundamentally different from conventional quantized signal processing approaches, which typically aim to minimize an expected error measure, such as the mean squared error. Based on the principle of maximizing the preserved relevant information under quantization, we presented and investigated an iterative receiver structure for a frequency-flat fading channel that employs advanced signal processing concepts for iterative LDPC decoding, decision feedback-aided channel estimation and detection. All signal processing units in this receiver were designed using the information bottleneck principle of maximizing the preserved relevant information under quantization. The corresponding signal processing operations applied in the constructed receiver were implemented as static lookup tables that were designed offline with the information bottleneck method. Despite this fact, the designed receiver did not suffer from any mentionable performance degradation in comparison with a conventional receiver with double-precision signal processing and belief propagation decoding.

After having studied the fundamental idea and the lookup table-based implementation of information bottleneck signal processing units in the receiver implementation, other methods for learning and implementing mutual information-maximizing signal processing units for LDPC decoders were also recalled from the literature and investigated. Our main conclusion is that the considered approaches to information bottleneck signal processing, including the one based on lookup tables, effectively aim to learn the parameters θ of trainable functions $f_{\theta }\left(y\right)$ that are tuned to maximize the preserved relevant information I$\left(X;f_{\theta }\left(Y\right)\right)$ under a constraint for the cardinality of the value set of $f_{\theta }\left(y\right)$. We note that the ways to learn the respective parameters differ depending on the kind of function that is designed to preserve the relevant information. While the lookup table based approaches typically employ information bottleneck algorithms, the computational domain approach from [24,25] uses a quantizer design algorithm to determine the optimum quantization thresholds. The neural network-based approaches from [28,29] mimic lookup tables or directly use gradient-based algorithms to maximize the preserved relevant information. Even more ideas that use nearest neighbor search algorithms to implement information bottleneck signal processing units and genetic algorithms to tune their parameters were proposed in the literature [64,65]. Finally, what all these approaches have in common is the learning of trainable parameters to maximize the preserved relevant information under quantization.

A lot of very interesting open research questions on information bottleneck signal processing arise from the problem of finding powerful and, at the same time, simple trainable functions for different signal processing applications that allow preserving huge amounts of relevant information. Moreover, exploring the methods for efficient training of the involved parameters, such as using genetic algorithms or gradient-based schemes, is interesting.

## Data Availability

Not applicable.
